# Hydroxychloroquine Alleviates EAU by Inhibiting Uveitogenic T Cells and Ameliorating Retinal Vascular Endothelial Cells Dysfunction

**DOI:** 10.3389/fimmu.2022.859260

**Published:** 2022-03-25

**Authors:** Yunwei Hu, Zuoyi Li, Guanyu Chen, Zhuang Li, Jun Huang, Haixiang Huang, Yanyan Xie, Qian Chen, Wenjie Zhu, Minzhen Wang, Jianping Chen, Wenru Su, Xiaoqing Chen, Dan Liang

**Affiliations:** State Key Laboratory of Ophthalmology, Zhongshan Ophthalmic Center, Sun Yat-sen University, Guangdong Provincial Key Laboratory of Ophthalmology and Visual Science, Guangzhou, China

**Keywords:** Hydroxychloroquine, experimental autoimmune uveitis, T cells, retinal vascular endothelial cells, lectin-like oxidized LDL receptor-1 (LOX-1), nuclear factor κB (NF-κB)

## Abstract

**Purpose:**

Inflammation triggers the activation of CD4^+^T cells and the breakdown of blood–retinal barrier, thus contributing to the pathology of experimental autoimmune uveitis (EAU). We explored the anti-inflammatory effect of hydroxychloroquine (HCQ) on EAU and the potential mechanisms active in T cells and retinal vascular endothelial cells (RVECs).

**Methods:**

C57BL/6J mice were immunized with interphotoreceptor retinoid binding protein 1-20 (IRBP_1–20_) to induce EAU and then treated with the vehicle or HCQ (100 mg/kg/day). On day 7, 14, 21, 30 and 60 after immunization, clinical scores were evaluated. On day 14, histopathological scores were assessed, and retinas, spleens, and lymph nodes were collected for quantitative polymerase chain reaction or flow cytometry analysis. RVEC dysfunction was induced by tumor necrosis factor α (TNF-α) stimulation. The expression of cytokines, chemokines, adhesion molecules, and lectin-like oxidized LDL receptor-1 (LOX-1)/nuclear factor κB (NF-κB) was measured in RVECs with or without HCQ.

**Results:**

HCQ treatment protected mice from uveitis, evidenced by reduced expression of inflammatory factors, chemokines, and adhesion molecules in the retina. In systemic immune response, HCQ inhibited the activation of naïve CD4^+^T cells and frequencies of T effector cells, and promoted T regulatory cells. HCQ decreased IRBP_1-20_–specific T cell responses and proliferation of CD4^+^T cells *in vitro*. Further studies established that TNF-α induced RVECs to express inflammatory cytokines, chemokines, and adhesion molecules, whereas HCQ alleviated the alterations *via* the LOX-1/NF-κB pathways.

**Conclusions:**

HCQ alleviates EAU by regulating the Teff/Treg balance and ameliorating RVECs dysfunction *via* the LOX-1/NF-κB axis. HCQ may be a promising therapeutic candidate for uveitis.

## 1 Introduction

Autoimmune uveitis (AU) is a chronic inflammatory disease affecting ocular tissues, including the retina and retinal blood vessels, and is the fifth most common cause of blindness worldwide (1). The socio-economic burden of AU is comparable to that of diabetic retinopathy (2). Glucocorticosteroids, immunosuppressants, and biologics are widely recognized as first-line treatment modalities for uveitis; however, the adverse effects and the high rate of unresponsiveness are concerning (3). Therefore, novel, safe, and effective treatments are urgently needed.

Experimental autoimmune uveitis (EAU) is a widely used model of T cell-mediated AU, which is induced by immunization with interphotoreceptor retinoid binding protein 1-20 (IRBP_1–20_) in an adjuvant context (4). It was reported that uveitogenic T cells, producing proinflammatory cytokines, and retinal vascular endothelial cells (RVECs), expressing adhesion molecules and chemokines to recruit immune cells, are responsible for retinal tissue damage. Thus, targeting both T cells and RVECs should be a promising treatment strategy for AU (4, 5).

Chloroquine (CQ) and its hydroxyl analog, hydroxychloroquine (HCQ), are low-cost antimalarial agents, with few side effects, and have been used for half a century. Besides, the immunomodulatory properties of these compounds allow them to protect against many inflammatory diseases, including rheumatoid arthritis, systemic lupus erythematosus (SLE), and multiple sclerosis (6, 7). In ophthalmic clinics, CQ/HCQ were reported to treat Graves’ orbitopathy by inhibiting proliferation, adipogenesis, and hyaluronan generation in orbital fibroblasts isolated from patients (8).

Some *in vitro* studies have shown that CQ/HCQ inhibit the production of tumor necrosis factors α (TNF-α), interleukin (IL)-1β, and IL-6 in lipopolysaccharide-stimulated human monocytes/macrophages and reduce the levels of proinflammatory cytokines in the serum of patients with SLE (9, 10). As the majority of previous studies focused on the innate immunity, only a few *in vitro* studies showed that HCQ could reduce the activation, proliferation and differentiation of T cells (11–13). However, molecular mechanisms underlying the anti-inflammatory effects of CQ/HCQ on T lymphocytes have not been clearly elucidated, and it is not known whether CQ/HCQ have therapeutic effects on uveitis.

CQ/HCQ have also been applied as adjuvant therapy for arteriosclerosis, antiphospholipid syndrome, preeclampsia, and pulmonary hypertension considering their endothelial cell protection ability (14–18). Moreover, they were reported to decrease the vascular oxidative stress in mice with lupus (19). To date, the mechanisms underlying the protective effects of HCQ on endothelial cell-mediated inflammatory events in autoimmune diseases are unclear.

In clinical practice, HCQ is preferred than CQ because of the lower incidence of gastrointestinal adverse reactions and decreased risk of ocular adverse events associated with the former. Therefore, we investigated the effects of HCQ in EAU to expand its application in ocular diseases, and explored its effects and potential mechanisms on T cells as well as on RVECs.

## 2 Materials and methods

### 2.1 EAU Induction and Treatment

#### 2.1.1 Animals

Female wild type C57BL/6J mice (6–8 weeks old) were purchased from Guangzhou Animal Testing Center and housed under specific pathogen-free conditions with a 12 h light-dark cycle, and stable temperature (23 ± 2°C) and relative humidity (55 ± 10%). All experimental animal protocols were approved by the Institutional Animal Care and Use Committee of Zhongshan Ophthalmic Center, Sun Yat-sen University, and all procedures were performed in compliance with the Association for Research in Vision and Ophthalmology (ARVO) Statement for the Use of Animals in Ophthalmic and Vision Research.

#### 2.1.2 EAU Induction by Active Immunization

Human IRBP_1-20_ (GPTHLFQPSLVLDMAKVLLD, Shanghai Shengong Company, China) was emulsified with complete Freund’s adjuvant (CFA) containing 5 mg/mL heat-denatured *Mycobacterium tuberculosis* (Chondrex, LLC, Seattle, WA) in a 1:1 volume ratio (v/v). C57BL/6J mice were injected subcutaneously with 200 µL emulsion (IRBP_1-20_ 200 µg/mouse) at the base of two thighs and on the back for immunization. Thereafter, each mouse received 200 ng of pertussis toxin (List Biological Laboratories, Campbell, CA) intraperitoneally on day 0 and day 2 after immunization.

#### 2.1.3 Treatment of EAU Mice

HCQ (CAS No. 747-36-4, Selleck Chemicals Co., Houston, USA) was dissolved in phosphate-buffered saline (PBS) solution as previously described (20). Mice recieved intraperitoneal injection of HCQ (100 mg/kg/day) from day 0 until day 30 or sacrifice. The control were injected PBS solution only.

#### 2.1.4 Clinical and Histopathologic Evaluation of EAU Retina Inflammation

On day 7, 14, 21, 30, and 60 after immunization, the fundus was photographed (Phoenix Co., Campbell, California, USA). On day 14, eyes were dissected, fixed, and stained with hematoxylin-eosin (HE). The clinical and pathological scores were graded on a scale from 0 to 4, as previously described (21).

### 2.2 Lymphocyte Isolation and Treatment

For *in vitro* assays, the cells were isolated from the dLNs and spleens of C57BL/6J mice. Lymphocyte suspensions were prepared by grinding and tapping the organs through a 70-μm nylon mesh, and then centrifuged at 1200rpm at 4°C for 5 minutes to get cell pellet. Red blood cells (RBCs) were removed using RBC lysis buffer (Biolegend) for 2 min. At last, lymphocytes were suspended in 10% FBS 1640 complete medium and counted for future use. *In vitro* studies, we evaluated the toxic effects of HCQ at different concentrations (0, 20, 40, 60, or 80 µM) on lymphocytes by flow cytometry with Zombie NIR™ Dye (Biolegend, San Diego, CA).

#### 2.2.1 T Cell Recall Assays

T cell recall assays were performed on day 7 after immunization. Cells were seeded into 96-well plates at a density of 2×10^5^ cells/well and cultured in complete RPMI-1640 medium (with 10% fetal bovine serum) supplemented with 20 µg/mL IRBP_1-20_ at 37°C and 5% CO_2_, and treated with HCQ at different concentrations (0, 20, 40, or 60 µM). After 72 h, the cells were harvested for flow cytometry.

#### 2.2.2 T Cell Proliferation Assay

The cells from naïve C57BL/6J mice were first labeled with Tag-it Violet™ Proliferation and Cell Tracking Dye (Biolegend, San Diego, CA, USA), and sorted using Dynabeads™ Untouched™ Mouse T Cells Kit (Invitrogen, California, USA) for getting CD3^+^ T cells. The labeled CD3^+^ T cells were activated with anti-CD3/CD28 beads (ratio 2:1) in 96-well plates at 2×10^5^ cells/well for 4 days with/without HCQ (20, 40, or 60 µM).

### 2.3 Human Retinal Vascular Endothelial Cells (HRVECs) and Treatment

#### 2.3.1 Cell Culture and TNF-α–Induced Inflammatory Dysfunction

Primary HRVECs were obtained from ScienceCell (Carlsbad, CA) and cultured with ECM medium (Zhong Qiao Xin Zhou Biotechnology Co., Ltd, Shanghai, China) containing growth factors and 5% FBS in a humidified incubator at 37°C with 5% CO_2_. Cell Counting Kit-8 (CCK-8) assay kit (Bimake, Shanghai, China) was used to measure the effects of HCQ on the cell viability of RVECs: RVECs were seeded in 96-well plates with HCQ (0, 20, 40, 80 and 100 µM) for 48 h. At that time, cells were added with CCK-8 reagent diluted at a 10:1 ratio and cultured for another 2 hours before the OD value of each well was measured at 450 nm using a Synergy H1 microplate reader (BioTek, Vermont, USA). Cells were pretreated with HCQ at nontoxic concentrations for 10 h at 80%–90% confluence, and then incubated with recombinant TNF-α (10 ng/mL, Life Technologies, Carlsbad, CA) for different time according to experimental requirements.

#### 2.3.2 Isolation of Peripheral Blood Mononuclear Cells and Coculture With RVECs

PBMCs of three uveitis patients were isolated immediately by density gradient centrifugation (Ficoll-Hypaque; Pharmacia Biotech, Shanghai, China). The study was performed in accordance with the tenets of the Declaration of Helsinki, and institutional review board approval. Written informed consent was obtained from each patient. RVECs were pretreated with HCQ at 0, 20, 40, 80 µM for 10 h in 96-well plates (2×10^3^ cells/well), and then incubated with recombinant TNF-α for another 14 h. The culture medium was changed to RPMI-1640 for coculture with lymphocytes. Fresh PBMCs (2×10^5^ cells/well) were added to 96-well plates and cocultured with RVECs for 48 h. The inflammatory cells were analyzed by flow cytometry.

### 2.4 Single-Cell RNA Sequencing Analysis Using a Public Database

The gene expression omnibus (GEO) database was searched and the single-cell sequencing dataset of spontaneous autoimmune uveitis, GSE132229, was selected. The cells subsets were grouped according to the annotation in original data. The transcripts were analyzed by R (4.0.3). The R package limma was employed to identify the differentially expressed genes (DEGs) with a fold change greater than 2 and p-value < 0.05. The up- or downregulated genes were assigned biological functions according to the Database for Annotation, Visualization, and Integrated Discovery (DAVID). Kyoto Encyclopedia of Genes and Genomes (KEGG) analysis was applied for pathway enrichment analyses, and gene set variation analyses (GSVA) were performed for the analysis of gene set enrichment. Enrichment score >1.0 and p-value <0.05 were set for the identification of gene ontology (GO) and GSVA terms.

### 2.5 Flow Cytometry

Flow cytometry was used for analyzing the toxic effects of HCQ on lymphocytes with Zombie NIR™ Dye (Biolegend, San Diego, CA), and the frequencies of naïve CD4+T (CD4+CD62L+CD44-), Th1 (CD4+IFN-γ+), Th17 (CD4+IL-17A+), T regulator (Tregs) (CD4+CD25+Foxp3+) cells, and the expression of LOX-1 in RVECs. The cells were incubated with Fc block (clone 2.4G2, Bio Xcell) and stained with following antibodies from BioLegend: anti-mouse CD3 (BV421), anti-human CD3 (BV421), anti-human CD8 (PE), anti-human LOX-1 (APC), anti-mouse CD4 (Percp-cy5.5), anti-mouse CD25 (PE-cy7), anti-mouse CD44 (APC), anti-mouse CD62L (FITC), and anti-mouse CD69 (PE). For intracellular cytokine staining, the cells were stimulated with phorbol 12-myristate 13-acetate (PMA) (50 ng/mL; Sigma), ionomycin (500 ng/mL; Sigma), and Brefeldin A (1 µg/mL; Sigma) for 4–5 h. The The intracellular cytokines or transcription factor were stained with anti-human/mouse IFN-γ (BV786), anti-human/mouse IL-17A (BV650), and anti-human/mouse Foxp3 (FITC) after fixation and permeabilization. Data were analyzed using the FlowJo software 10.0 (Tree Star, Ashland, OR, USA).

### 2.6 Gene Expression by Real-Time PCR

The retinal tissues were isolated from vehicle- and HCQ-treated EAU mice. RVECs pretreated with/without HCQ for 10 h were incubated with TNF-α for another 14 h for RNA isolation. Total RNA was isolated using the RNeasy Mini kit (Qiagen Inc., Valencia, CA), and transcribed into cDNA using the HiScript II Q RT SuperMix for qPCR (Vazyme, Nanjin, China). Real-time quantitative PCR (qRT-PCR) was conducted using ChamQ SYBR^®^ qPCR Master Mix (Vazyme, Nanjing, China), according to the manufacturer’s instructions. The relative expression of IL-17A, IFN-γ, IL-1β, CXCR2, CXCR10, CXCR11, CD74, monocyte chemotactic protein 1 (MCP-1), vascular cell adhesion molecule-1 (VCAM-1), intracellular adhesion molecule-1 (ICAM-1), E-selectin, lectin-like oxidized low-density lipoprotein receptor 1 (LOX-1), nuclear factor κB (NF-κB), and matrix metalloproteinase-9 (MMP-9) was analyzed using the comparative CT (ΔΔCT) method.

### 2.7 Western Blot Analysis

RVECs pretreated with/without HCQ for 10 h were incubated with TNF-α for another 24 h for western blot analysis. Cells were lysed with lysis buffer on ice for 15 min, and then centrifuged for 10 min at 12,000 r.p.m. and 4°C to harvest the supernatant. The protein concentrations were measured by a BCA protein assay (Pierce, Rockford, IL, USA). Supernatants were incubated with 5× Laemmli sample buffer (ThermoFisher, Waltham, Mass, USA) at 100°C for 8 min. Equal amounts of samples were then separated on SDS-PAGE gel, transferred on nitrocellulose membranes, and immunoblotted with NF-κB p65, phospho-NF-κB p65 (Ser536), and GAPDH antibodies (Cell Signaling Technology, Danvers, Massachusetts, USA).

### 2.8 Immunofluorescence Staining

RVECs seeded in 24-well plates were pretreated with/without HCQ for 10 h and incubated with TNF-α for another 24 h at 80%–90% confluency. After stimulation, cells were fixed with 4% paraformaldehyde for 30 min, washed with PBS, permeabilized with 0.3% Triton X-100 for 20 min, and blocked with 3% BSA for 2 h at room temperature. Subsequently, they were stained overnight with rabbit anti-phospho-NF-κB p65 (Ser536) (Cell Signaling Technology, Danvers, Massachusetts, USA) at 4°C, washed with PBS, incubated with Alexa 488-conjugated goat anti-rabbit antibody (Invitrogen, California, USA) for 2 h, and then with DAPI (Abcam, Cambridge, UK) for 10 min. Cells were imaged using an inverted fluorescent microscope (Nikon).

### 2.9 Statistical Analysis

Data were displayed as mean ± SD and statistically analyzed using a Student’s *t*-test (for parametric data) or Mann–Whitney U test (for nonparametric data) for two-group comparisons. A *p*-value <0.05 was considered statistically significant.

## 3 Results

### 3.1 HCQ Alleviates Retinal Inflammation in EAU

**Figure 1:**
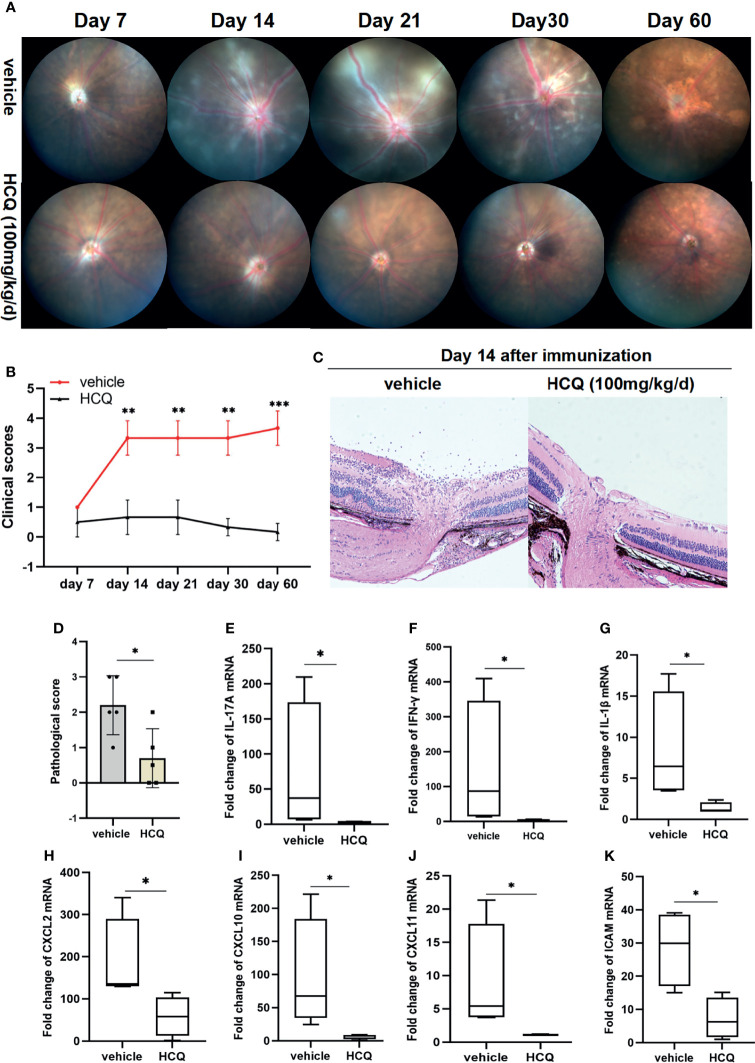
Hydroxychloroquine (HCQ) alleviates retinal inflammation in experimental autoimmune uveitis (EAU). C57BL/6J mice immunized with hIRBP1-20 were treated with HCQ (100 mg/kg) or vehicle daily. **(A, B)** Representative photos of fundus showed attenuation of retinal inflammation by HCQ, characterized by fewer linear lesions and minimal vasculitis of the retina. HCQ had persistent anti-inflammatory effects on EAU till day 60 after immunization. The clinical scores of EAU in the two groups were evaluated and compared. (N=5). **(C, D)** The eyes were enucleated and prepared for staining. Representative histopathological images showing that HCQ considerably reduced the structural damages. The histopathological scores were statistically different (N=5). **(E–K)** RT-qPCR results showed that the expression of IL-17A, IFN-γ, IL-1β, CXCL2, CXCL10, CXCL11, and ICAM mRNAs in retina was upregulated in uveitic retinas, whereas HCQ treatment considerably decreased the mRNA expression of these genes (N=4). The data are presented as the mean ± SD (*P < 0.05, **P < 0.01, ***P < 0.001).

To examine the therapeutic effects of HCQ on EAU, mice were injected intraperitoneally with HCQ (100 mg/kg/day). No abnormality such as weight loss, lethargy, or depilation was observed in any of the mice. Clinical changes were evaluated at day 7, 14, 21, 30 and 60 after IRBP_1-20_ immunization. Representative images of fundus demonstrated diffuse inflammatory lesions, severe vascular leakage, and dilation of blood vessels in vehicle-treated group. Retinal inflammation began to decrease with scar formation after day 30 and the atrophy of the retinas were presented at day 60. However, EAU mice treated with HCQ exhibited an almost normal retina or slight papilloedema. HCQ alleviated ocular manifestations pronouncedly and had persistent anti-inflammatory effects on EAU (**Figures 1A, B**). Histopathologically, uveitic pathology (Day 14 after immunization) was revealed by massive inflammatory cell infiltration, retinal swelling and folds, and moderate structural damage. Compared with vehicle group, slight inflammatory injuries were seen in HCQ group (**Figures 1C, D**).

We analyzed the expression of inflammatory genes in retinal tissues in both groups. Significant increase in IL-17A, IFN-γ, and IL-1β expression was detected in EAU mice, whereas HCQ suppressed the expression of these inflammatory genes (**Figures 1E–G**). Uveitogenic T cells migrate across the disrupted blood–retinal barrier (BRB) and gather in retinal tissue, resulting in directly ocular damage. Besides inflammatory cytokines, chemokines facilitate a highly directed migration of effector T cells to inflammatory lesions. Chemokine ligands and adhesion molecules expressed in retinal tissues contribute greatly to lymphocyte recruitment and are indispensable for uveitis. We assayed the mRNA expression of chemokines and found an augmentation of CXCL-2, CXCL-10, CXCL-11, and ICAM-1 mRNA expression in retinas of mice with EAU whereas, they were downregulated significantly in HCQ-treated mice (**Figures 1H–K**). Altogether, these results demonstrated that HCQ significantly alleviated the severity of retinal inflammation in EAU by suppressing the expression of inflammatory genes, and restraining relevant chemokines and adhesion molecules for lymphocyte recruitment.

### 3.2 HCQ Maintains Systemic Teff/Treg Balance to Ameliorate EAU

Uveitis is a T cell-mediated autoimmune disease, and the spleen and draining lymph nodes are important organs in initiation and regulation of immune responses in uveitis. After encountering antigens, naïve T cells in spleens and dLNs can differentiate into effector T cells (Teffs), including Th1 and Th17 cells, which contribute to ocular inflammation. In the meantime, naïve T cells can polarize into regulator T cells (Tregs), which possess suppressive effects to confront Th1/Th17 cells.

**Figure 2:**
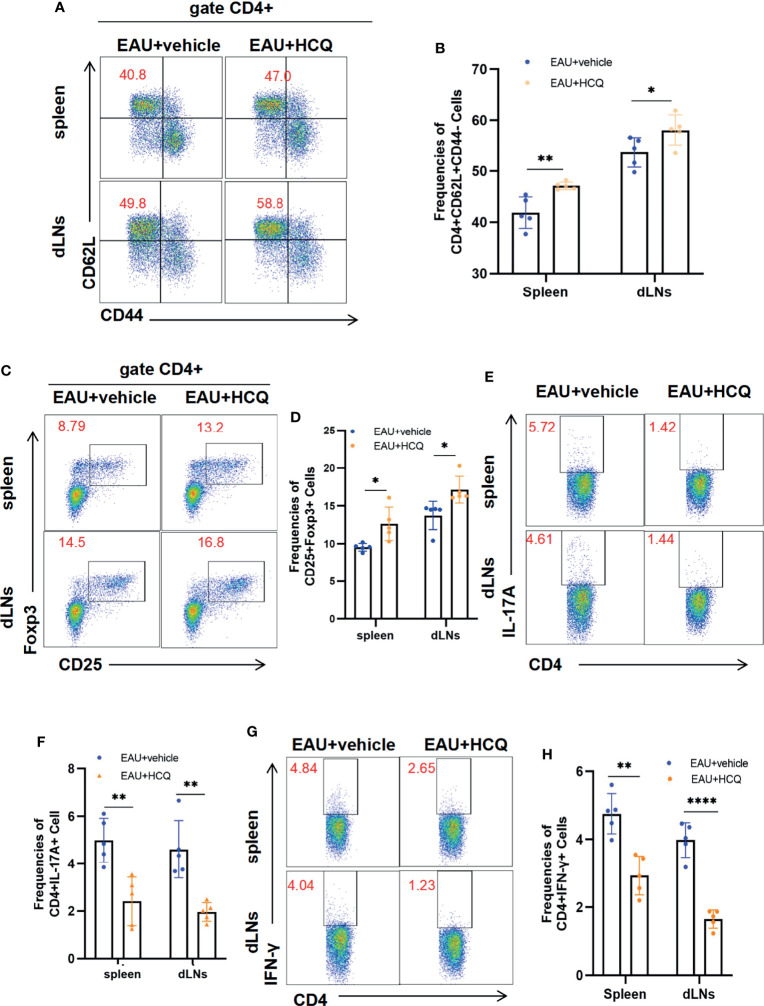
Hydroxychloroquine (HCQ) maintains systemic Teff/Treg balance to ameliorate experimental autoimmune uveitis (EAU). The lymphocytes were isolated from the spleens and draining lymph nodes (dLNs) of C57BL/6J mice on day 14 after immunization (N=5). Flow cytometry experiments were gated on CD4^+^ cells. **(A, B)** HCQ inhibited the naïve CD4^+^T cell (CD62L+CD44-) differentiation into memory CD4^+^T cells (CD62L-CD44+) compared with EAU treated with the vehicle, in both spleens and dLNs. **(C, D)** The proportion of Treg cells in spleens and dLNs was promoted by HCQ administration in EAU mice. **(E‐H)** HCQ decreased the frequencies of Th1 and Th17 cells in spleens and dLNs. The data are presented as the mean ± SD. (*P < 0.05, **P < 0.01, ****P < 0.0001).

Hence, we explored the effects of HCQ on naïve T cell activation and Teff/Treg balance in peripheral immune organs of EAU. HCQ-treated EAU mice had more naïve cells (CD4+CD62L+CD44-) in spleens and dLNs than those in vehicle group (**Figures 2A, B**). Flow cytometry results showed that HCQ treatment increased the frequency of Tregs (**Figures 2C, D**) and decreased the frequencies of Th1 and Th17 cells (**Figures 2E–H**) in spleens and dLNs compared with those in the vehicle group. These findings indicated that HCQ prevented Teff/Treg imbalance by increasing Tregs and decreasing Teff in spleens and dLNs of EAU mice.

### 3.3 HCQ Inhibits the Differentiation and Proliferation of T Cells

**Figure 3:**
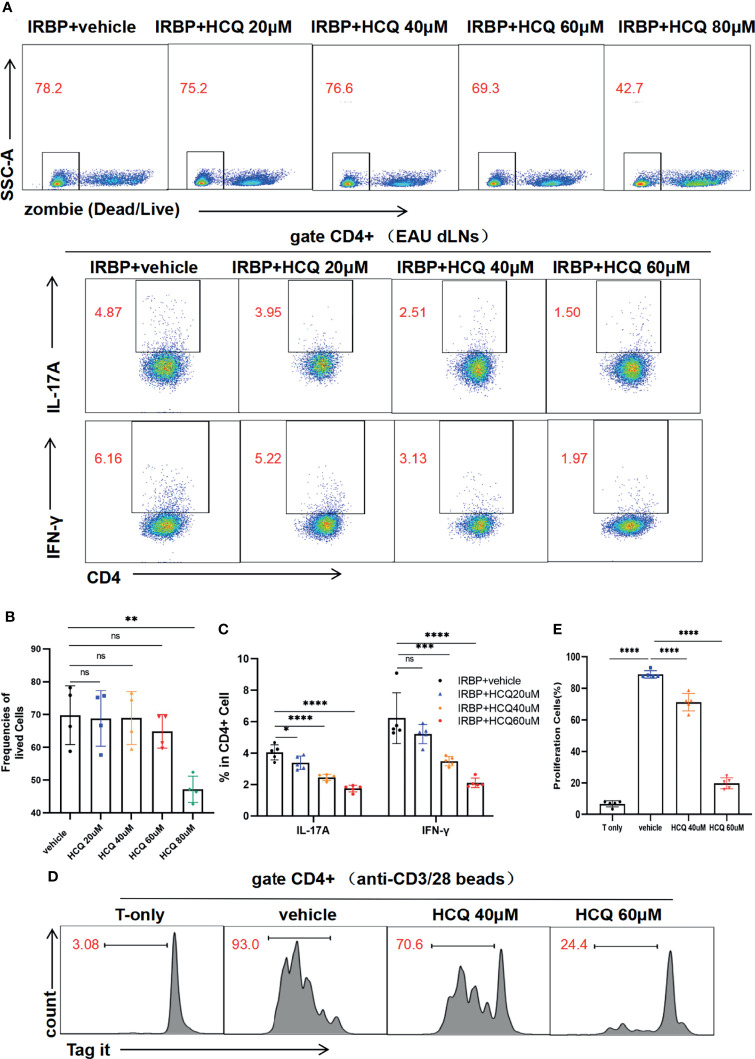
Hydroxychloroquine (HCQ) suppresses the differentiation and proliferation of T cells. **(A–C)** The lymphocytes from draining lymph nodes (dLNs) of experimental autoimmune uveitis (EAU) mice were isolated and cultured *in vitro* with hIRBP_1-20_ for 3 days. The percentages of viable lymphocytes (zombie-) were similar at concentrations of 0, 20, 40, and 60 μM of HCQ, whereas 80 μM HCQ treatment decreased the percentage. HCQ (40 and 60 µM) suppressed the differentiation of Th17 and Th1 cells significantly with statistical differences, and 20 µM HCQ suppressed Th17 differentiation similarly (N= 5). **(D, E)** The labeled T cells were sorted and activated with anti-CD3/CD28 beads for 4 days. HCQ (40 and 60 µM) inhibited the proliferation of CD4^+^T cells obviously with statistical difference (N=5). The data are presented as the mean ± SD (^ns^P > 0.05, *P < 0.05, **P < 0.01, ***P < 0.001, ****P < 0.0001).

Retinal self-antigen-specific T cells cause inflammation and tissue damage in EAU. To address whether HCQ could shape IRBP_1-20_–specific Teff *in vitro*, we collected lymphocytes from the dLNs of EAU mice and stimulated them with IRBP_1-20_ in the presence of HCQ at different concentration gradients. The flow cytometry results showed that cell viabilities were similar at the concentration of 0, 20, 40 and 60 μM, whereas at 80 μM, HCQ decreased the viability of the cells greatly (**Figures 3A, B**). Therefore, HCQ at the concentrations of 20/40/60μM were used for lymphocytes in this study. Three days later, we analyzed the proportions of Th1/Th17 cells to assess T cell differentiation. Compared with IRBP+vehicle group, HCQ reduced the frequencies of Th17 and Th1 cells notably in a concentration-dependent manner (**Figures 3A, C**). Conclusively, HCQ inhibited the IRBP_1-20_–specific T cell immune responses by restraining Th1/Th17 cells.

Besides, T cell activation and differentiation is often accompanied with proliferation. We analyzed the proliferation of T cells in the presence or absence of HCQ. CD4^+^T cells were sorted and labeled, and then cultured with anti-CD3/28 bead stimulation for 4 days. HCQ could inhibit CD4^+^T cell proliferation at 40 and 60 µM (**Figures 3D, E**). Taken together, these results suggested that HCQ could inhibit T cell differentiation and proliferation *in vitro*.

### 3.4 Analysis of Alteration of RVECs in Uveitis Using a GEO Dataset

**Figure 4:**
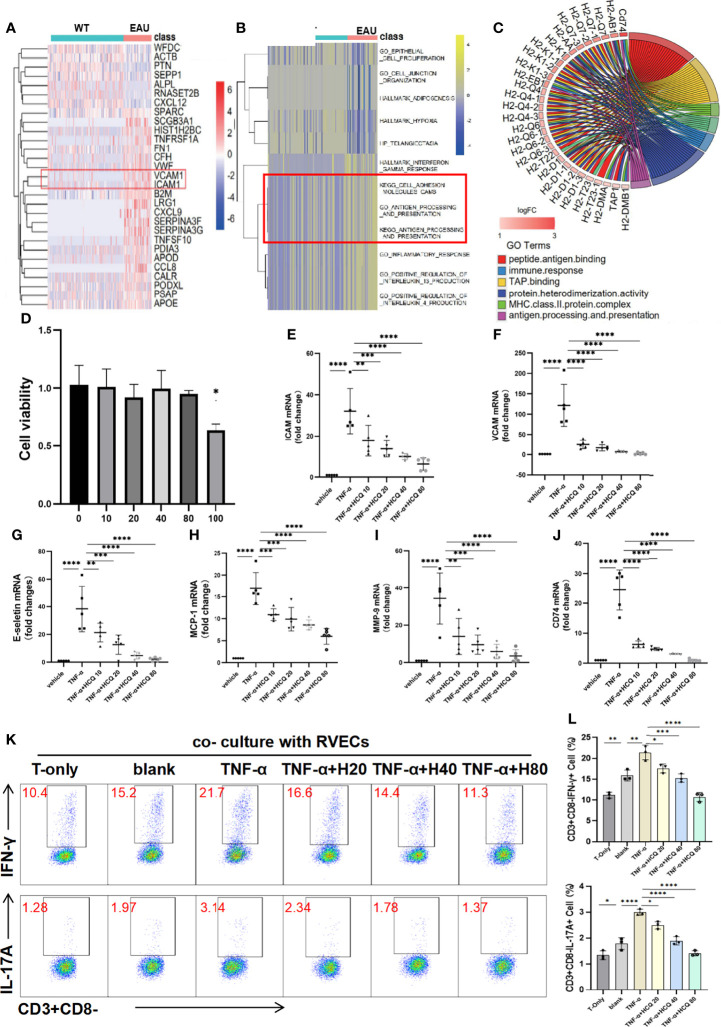
Hydroxychloroquine (HCQ) alleviates retinal vascular endothelial cells (RVECs) dysfunction induced by inflammation. **(A)** Differentially expressed genes (DEGs) were explored and are reported in a heatmap of mRNA abundance. Rows denote expression of RNAs according to their enrichment in WT and spontaneous experimental autoimmune uveitis (EAU). **(B)** The heatmap of the gene ontology (GO) and Kyoto encyclopedia of genes and genomes (KEGG) analyses showed that compared with the WT group, uveitic RVECs presented with enhanced inflammatory response, cell adhesion molecules CAMS, and antigen processing and presentation. **(C)** Chord chart equally illustrates that the antigen presentation mediated by MHC-II molecules was significantly enhanced in RVECs of EAU mice. The inflammatory dysfunction of RVECs was induced by TNF-α stimulation. **(D)** The toxic effects of HCQ on the cell viability of RVECs were measured by CCK8 assay. RVECs were incubated with 10, 20, 40, 80 and 100 μM HCQ for 48 h, and 100 μM HCQ induced cytotoxicity on RVECs (N=3). **(E–J)** RT-qPCR results show that in the model of RVEC dysfunction, the expression of ICAM, VCAM, E-selectin, MCP-1, MMP-9, and CD74 mRNAs in the retina was markedly upregulated, whereas HCQ (10, 20, 40, and 80 µM) considerably and dose-dependently decreased the expression of these mRNAs (N=5). **(K, L)** Flow cytometry analysis shows that the T cells were activated and produced more IL-17 and IFN-γ after coculture with RVECs, especially with TNF-α–stimulated RVECs. Pretreatment of TNF-α–stimulated RVECs with HCQ resulted in lower cytokine production in T cells (N=3). The data are presented as the mean ± SD (*P < 0.05, **P < 0.01, ***P < 0.001, ****P < 0.0001).

To explore the alteration of RVECs in uveitic retinas, we searched the GEO database for RNA-seq datasets of RVECs in uveitis. The single-cell sequencing dataset of spontaneous autoimmune uveitis, GSE132229, was selected. Cell clusters generated from the t-SNE dimensional reduction were classified and annotated in original data. DEG analysis unravel that cytokines, chemokines, and adhesion molecules, including ICAM-1 and VCAM-1, were highly expressed in RVECs in uveitis (**Figure 4A**). The GO and KEGG analysis demonstrated that the inflammatory response, cell adhesion molecules, and antigen processing and presentation were enhanced in RVECs in uveitis (**Figure 4B**). It also illustrated that in uveitis, the antigen presentation mediated by major histocompatibility complex (MHC)-II molecules was significantly enhanced in RVECs (**Figure 4C**).

### 3.5 HCQ Attenuates TNF-α–Induced RVEC Inflammation

In pathological conditions, massive production of inflammatory cytokines triggers dysfunction of RVECs. Substantial evidence has revealed that TNF-α is the major proinflammatory factor involved in various pathogenesis. Therefore, the inflammatory dysfunction cell model of RVECs was established by TNF-α stimulation in culture.

As observed in bioinformatics analysis, dysfunctional RVECs exhibited upregulated endothelial adhesion molecules and chemokines, leading to increased adhesion and trans-endothelial migration of leukocytes. Then we conducted *in vitro* experiments to validate these findings and investigated the protective effects of HCQ on RVECs. The CCK8 assay indicated that when the concentration of HCQ was less than 80 μM, HCQ did not exert a significant impact on the cell viability of RVECs. However, 100 μM HCQ induced cytotoxicity on RVECs (**Figure 4D**). Therefore, HCQ at the concentrations of 10/20/40/80μM were used for RVECs. It was verified in the RVEC dysfunction model that the expression of VCAM-1, ICAM-1, and E-selectin mRNAs was highly upregulated after TNF-α stimulation, whereas HCQ treatment reversed the trend in a concentration-dependent manner (**Figures 4E–G**). Moreover, the mRNA expression of MCP-1 and MMP-9, was increased in TNF-α–stimulated RVECs, whereas HCQ significantly inhibited the expression of these genes (**Figures 4H, I**).

The antigen presentation ability of RVECs was predicted by bioinformatic analysis to be enhanced under TNF-α stimulation, characterized by highly expressed CD74, presuming closer interactions between RVECs and T cells. We verified that TNF-α induced CD74 on RVECs, and HCQ treatment reduced the expression of CD74 mRNA (**Figure 4J**). Furthermore, we cocultured RVECs with T cells, and analyzed the production of cytokines by T cells to explore the effect of HCQ on the interaction between RVECs and T cells. Flow cytometry revealed that coculture with RVECs activated T cells and increased cytokine production, especially with TNF-α–stimulated RVECs. However, HCQ treatment of TNF-α–stimulated RVECs resulted in lower cytokine production by T cells (**Figures 4K, L**). HCQ was found to relieve TNF-α–induced inflammatory dysfunction of RVECs and lessened the interaction between RVECs and T cells, further inhibiting T cell functions.

### 3.6 Effect of HCQ on TNF-α–Stimulated Activation of LOX-1/NF-κB Inflammatory Pathways in RVECs

LOX-1 is a membrane protein widely expressed on vascular endothelial cells. It belongs to the superfamily of scavenger receptors capable of binding TNF-α. The high expression of LOX-1 induced by TNF-α is associated with endothelial dysfunction (22). NF-κB is also a critical modulator of inflammation, and is possible to be activated through TNF-α pathway. Whether the RVEC dysfunction stimulated by TNF-α was mediated by LOX-1 upregulation and subsequent activation of NF-κB remains unknown. To investigate the mechanisms of regulation of RVECs dysfunction by HCQ, we evaluated the effect of HCQ on TNF-α–stimulated LOX-1/NF-κB signaling pathway activation.

**Figure 5:**
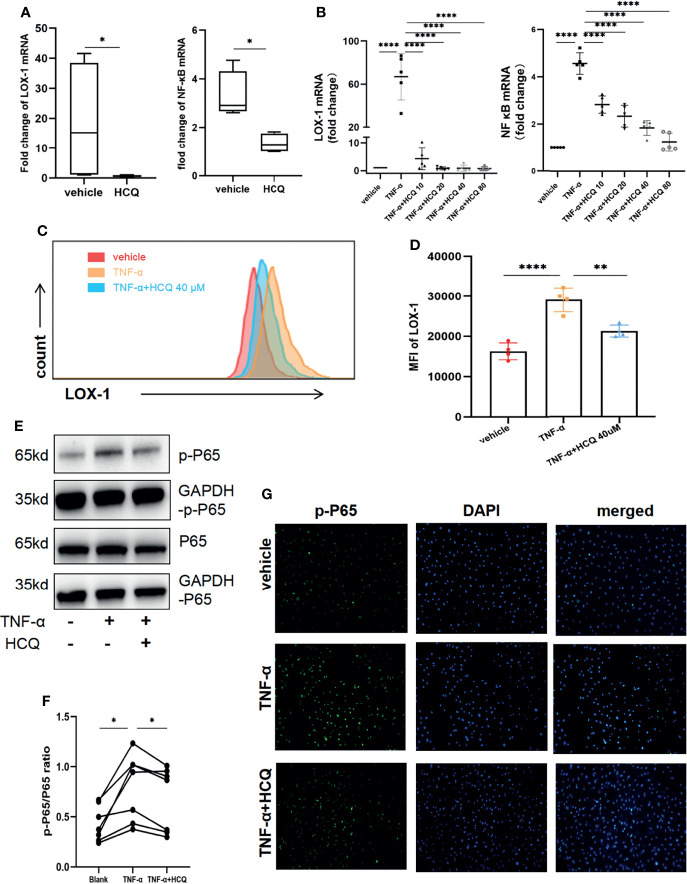
Hydroxychloroquine (HCQ) regulates TNF-α–stimulated endothelial dysfunction *via* the LOX-1/NF-κB axis. **(A)** In the *in vivo* study, RT-qPCR results showed that HCQ suppressed the LOX-1 and NF-κB mRNA expression in retinal tissue of experimental autoimmune uveitis (EAU) mice (N = 4). **(B)** In the *in vitro* studies, RT-qPCR revealed that HCQ (10, 20, 40, and 80 µM) considerably and dose-dependently decreased LOX-1 and NF-κB mRNA expression in the retinal vascular endothelial cell (RVEC) dysfunction model. Furthermore, we verified these results at the protein level (N = 5). **(C, D)** Flow cytometry analysis showed that the mean fluorescence intensity (MFI) of LOX-1 in TNF-α–stimulated RVECs was higher than that in the vehicle group. HCQ reduced its MFI at a concentration of 40 µM with a significant difference(N = 4). **(E, F)** Western blot analysis showed upregulated expression of total p65 (t-p65) and phosphorylated p65 (p-p65) in TNF-α–stimulated RVECs compared with those in the vehicle group. HCQ (40 µM) treatment suppressed their expression and lowered the p-p65/t-p65 ratio (N = 7). **(G)** Immunofluorescent staining showed colocalization of p-p65 (green) and the nuclei (blue) of RVECs. Elevated expression of p-p65 was observed in RVECs after TNF-α stimulation, whereas HCQ treatment reduced the expression of p-p65 in RVECs. The data are presented as the mean ± SD (*P < 0.05, **P < 0.01, ****P < 0.0001).

*In vivo*, we analyzed the LOX-1 and NF-κB gene expression in retinal tissues in EAU mice treated with/without HCQ. RT-qPCR displayed significant increase in LOX-1 and NF-κB expression in EAU mice, whereas HCQ administration suppressed the expression of both genes (**Figure 5A**). For *in vitro* studies, RVECs were pretreated with HCQ (40 μM) for 10 h and then stimulated with TNF-α (10 ng/mL) for another 14 h. Total RNA of RVECs was isolated for qPCR to measure the mRNA expression of LOX-1/NF-κB. LOX-1/NF-κB signaling pathways in TNF-α–stimulated RVECs was activated at the mRNA level, which were reduced by HCQ in a concentration-dependent manner (**Figure 5B**). Flow cytometry analysis presented that LOX-1 expression (mean fluorescence intensity, MFI) of TNF-α-stimulated RVECs was higher comparing to the vehicle group. Likewise, HCQ treatment reduced LOX-1 levels (**Figures 5C, D**). As p65 is a major subunit of NF-κB, we measured total p65 (t-p65) and phosphorylated p65 (p-p65) levels by WB analysis. It showed HCQ treatment inhibited phosphorylation of p65 subunit, resulting in a lower ratio of p-p65/t-p65 (**Figures 5E, F**). In addition, immunofluorescent staining showed colocalization of p-p65 and the nuclei of RVECs. Consistent with the former results, elevated expression of p-p65 was observed in TNF-α–stimulated RVECs, whereas HCQ attenuated this effect (**Figure 5G**). These data suggested an essential role of HCQ in regulating TNF-α–stimulated endothelial dysfunction, LOX-1 expression, and NF-κB inhibition.

## 4 Discussion

In this study, we explored the efficacy of HCQ on uveitis and its potential mechanism using an EAU model. HCQ alleviated the severity of retinal inflammation in EAU mice, reducing the inflammatory gene expression, as well as relevant chemokines and adhesion molecules for lymphocyte recruitment. In systemic immune response, HCQ inhibited the activation of naïve CD4^+^T cells, and maintained Teff/Treg cell balance by upregulating the frequencies of Tregs and downregulating that of Th1/Th17 cells. HCQ administration decreased IRBP_1-20_ specific T cell responses and inhibited proliferation of CD4^+^T cells *in vitro*. Furthermore, single-cell RNA sequencing and molecular mechanism studies established that RVECs exhibited high expression of inflammatory factors, chemokines, and adhesion molecules in the pathological state, whereas HCQ alleviated the alterations by regulating LOX-1/NF-κB signaling pathways. We cocultured RVECs with T cells, and analyzed the production of cytokines by T cells to found HCQ lessened the interaction between RVECs and T cells, with potential of inhibiting the recruitment of lymphocytes and T cell functions to mitigate EAU.

HCQ has been used for the treatment of many autoimmune diseases and improves the prognosis of the disease and lowers the levels of inflammatory factors in patients (23–27). Here, we proved that HCQ could alleviate the retinal inflammation and maintain the balance of Teff/Treg cells in systemic immune system in EAU mice, which provided a promising prospect and basis to expand the clinical application of HCQ to patients with autoimmune uveitis. Besides anti-inflammatory effect, HCQ originally were prescribed for prevention or treatment of malaria, and evaluated for various viral infections from HIV to COVID-19 recently, which lowered the risk of infections due to systemic suppression of immunity (28). In addition, HCQ had antithrombotic and antineoplastic effects, making it a potentially valuable treatment for patients with systemic autoimmune disorders who were at risk of malignancy and thrombotic events (29). During our animal studies, the mice administrated with HCQ were grown and feed healthy. HCQ has a good safety profile though caution was advised when using higher than usual doses. Although some previous reports mentioned the destructive effects of HCQ in maculopathy, it is now clear that its correct application rarely causes eye damages (30, 31).

In terms of cellular and molecular mechanisms underlying the anti-inflammatory effects, HCQ has been reported to inhibit the antigen presentation responses and TLR activation, as well as functions of macrophages/monocytes (32–35). However, uveitis is a T cell-mediated autoimmune disease, and autoreactive CD4^+^T cells play a major role in the initiation and orchestration of EAU. Direct targeting of T cells is key to the treatment of uveitis. Therefore, we focused on and revealed the inhibitory effects of HCQ on T cells, characterized by reducing Th1/Th17 differentiation, and restraining CD4^+^T cell activation and proliferation. Goldman et al. reported that HCQ exerted its immunomodulatory properties by inhibiting the TCR-crosslinking-dependent calcium signaling, which induced upregulation of CD69 expression, using a T cell line (12). Furthermore, Yang et al. found that HCQ reduced the differentiation of Th17 cells and the production of IL-17A *in vitro* using PBMCs from SLE patients (13). However, in both these studies no systematic animal experiments were conducted; our results consummate and validate their findings.

Another pivotal mechanism for autoimmune uveitis is the recruitment and migration of circulating antigen-specific leucocytes into retinal tissue through the expression of cellular adhesion molecules and break down of BRB (4). RVECs play an indispensable role in this pathologic process for interaction with pathogenic cells, especially T cells. Thus, we investigated the alterations in RVECs under inflammatory conditions using a public scRNA-Seq database and the TNF-α–stimulated RVEC dysfunction model. The bioinformatic analysis of scRNA-Seq hinted that RVECs have stronger antigen presentation and inflammatory response, as well as higher expression of cytokines, chemokines, and adhesion molecules in the spontaneous uveitis mouse model. Our *in vivo* and *vitro* studies confirmed the results and further demonstrated that HCQ could reduce the expression of cytokines, chemokines, and adhesion molecules in uveitic retinas and TNF-α–stimulated inflammation of RVECs. Our findings are in accordance with previous studies on other kinds of endothelial cells, which represent the pathophysiology of different diseases, like atherosclerosis, coronary heart disease, rheumatoid arthritis, and diabetes mellitus (36, 37). To conclude, the above results suggest that HCQ may be a promising molecule for treatment of inflammatory vascular diseases owing to its immunomodulatory effects.

TNF-α–stimulated inflammation is controlled by several signaling pathways. Among them, NF-κB pathway is critical for modulating the expression of a series of inflammation-related genes. Extracellular stimuli coupled with its receptors and lead to the rapid degradation of IkappaB (IκB), which facilitates translocation of NF-κB to the nucleus for regulation of gene transcription (38). It was proved that LOX-1 could be internalized into the cytoplasm and activated several cellular pathways, including the NF-κB signaling pathways, in vascular endothelial cells and TNF-α could be the trigger (22). Here, we observed the simultaneously elevated LOX-1 and NF-κB pathway in inflammatory retinas and RVECs, which contribute to increased inflammatory response, antigen presentation response, expression of cytokines, chemokines, and adhesion molecules. HCQ also reduced the expression of LOX-1 and NF-κB at both protein and RNA levels. Hence, we demonstrate that HCQ alleviates inflammtory dysfunction of RVECs *via* the LOX-1/NF-κB axis.

## 5 Conclusions

We show the therapeutic effects of HCQ against uveitis. HCQ regulates the Teff/Treg balance and ameliorates RVEC dysfunction *via* the LOX-1/NF-κB signaling pathways. These results expand our knowledge of the anti-inflammatory effects of HCQ and enrich our understanding of the pathogenic mechanism of EAU.

## Data Availability Statement

Datasets presented in this study can be found in online repositories. The names of the repository/repositories and accession number(s) can be found in the article/supplementary material.

## Ethics Statement

The studies involving human participants were reviewed and approved by The Ethics Committee of Zhongshan Ophthalmic Center, Sun Yat-sen University. The patients/participants provided their written informed consent to participate in this study. The animal study was reviewed and approved by The Institutional Animal Care and Use Committee of Zhongshan Ophthalmic Center, Sun Yat-sen University.

## Author Contributions

YH, ZuL, and GC were responsible for the conception and design of the study, experiments, data collection, data analysis, and manuscript writing. JH, ZhL, and HH helped in the *in vivo* experiment design and performance. WZ, MW, and JC helped in the *in vitro* experiment design and performance. YX, QC, and WSwere helpful in data analysis and manuscript writing. XC, and DL were responsible for conception and design, revision of the manuscript, and final manuscript approval. All authors contributed to the article and approved the submitted version.

## Funding

This work was supported by grants from the National Science Foundation of China (81870649, Guangzhou, Guangdong, China).

## Conflict of Interest

The authors declare that the research was conducted in the absence of any commercial or financial relationships that could be construed as a potential conflict of interest.

## Publisher’s Note

All claims expressed in this article are solely those of the authors and do not necessarily represent those of their affiliated organizations, or those of the publisher, the editors and the reviewers. Any product that may be evaluated in this article, or claim that may be made by its manufacturer, is not guaranteed or endorsed by the publisher.
